# COVID-19: imbalanced cell-mediated immune response drives to immunopathology

**DOI:** 10.1080/22221751.2022.2122579

**Published:** 2022-10-09

**Authors:** Jun Wang, Qian Li, YuanWang Qiu, Hongzhou Lu

**Affiliations:** a Department of Infectious Diseases, National Clinical Research Center for Infectious Diseases, Shenzhen Third People’s Hospital and The Second Affiliated Hospital of Southern University of Science and Technology, Shenzhen, People’s Republic of China; b Clinical Laboratory, The Fifth People’s Hospital of Wuxi, Jiangnan University, Wuxi, People’s Republic of China; c Department of Hepatology, The Fifth People’s Hospital of Wuxi, Jiangnan University, Wuxi, People’s Republic of China

**Keywords:** COVID-19, SARS-CoV-2, NETs, pathogenesis, cytokine storm

## Abstract

The coronavirus disease 2019 (COVID-19) pandemic, caused by severe acute respiratory syndrome coronavirus 2 (SARS-CoV-2), poses an imminent threat to humanity. SARS-CoV-2 invades host cells, causing a failure of host immune recognition. Instead of an effective antiviral immunological response after SARS-CoV-2 invasion, the cascading pathological syndrome of COVID-19, especially in severe disease, is exacerbated by an overt inflammatory response and the suppression of SARS-CoV-2–specific immune responses. As is known, excessive inflammation leads to pathophysiological changes in virus-infected tissues or organs, manifested by imbalanced immune responses, cytokine storm, and aggressive neutrophil activation, ultimately leading to lung damage, such as alveolar damage, endotheliitis, and fluid overload. However, the triggers and consequences of a disruption to immune system homeostasis and the underlying mechanisms of uncontrolled immunopathology following viral infection remain unclear. Here, we review the dynamic and systemic immune progression from an imbalance in cell-mediated immune responses to COVID-19 lung injury. Our understanding of key mechanisms involved in pathogenesis is critical for the development of therapeutic agents and to optimize therapeutic strategies.

## Introduction

The coronavirus disease (COVID-19) pandemic remains to be a serious public health emergency and economic crisis [1]. As of 6 May 2021, 256,966,237 COVID-19 confirmed cases and 5,151,643 deaths in >100 countries around the world have been documented [2]. Severe acute respiratory syndrome coronavirus 2 (SARS-CoV-2), the highly transmissible and pathological factor of COVID-19, together with the emerging variants, leads to a variety of symptoms ranging from upper respiratory tract compromise to severe pneumonia among different individuals with distinct host immunities [3].

Despite great efforts directed toward achieving herd immunity and infection control, such as public health interventions, as well as the rapid development and application of vaccines and pharmaceuticals worldwide, the pandemic remains a vexing concern since host immunity varies among individuals and populations. At the early stage of this pandemic, neutrophilia, and lymphopenia in COVID-19 patients, especially in severe cases, were quickly noticed by scientists. The neutrophil–lymphocyte ratio (NLR) was subsequently designated a useful factor to reflect the severity of pneumonia [4,5]. In addition, early analysis of kinetic changes in lymphocytes indicated that CD8^+^T-cell counts were dramatically reduced in severe COVID-19 patients [6].

Other than the changes in cell composition, many researchers grew interests in the key factors of the imbalanced host immune response and the pathogenesis of lung injury in COVID-19 [7]. Previous research on whether SARS-CoV-2 could induce strong IFN response were apparently controversial. It was finally censused that early IFN response fails to clear SARS-CoV-2 infection, while a partially elevated production of interferons (IFNs) and the expression of Interforon-stimulated genes (ISGs) were found in moderate and severe COVID-19 patients [8]. Moreover, another one of our publications clarified neutrophil extracellular trap (NET)-associated gene activation by analyzing BALF and PBMCs from COVID-19 patients [5]. These findings implying that the delayed IFN response and NET formation are key drivers of the progression of COVID-19 [5,9,10]. However, the details of the dynamic progression from an imbalanced immune response triggered by SARS-CoV-2 infection to COVID-19 lung injury remain unclear.

Better insight into the immunological and immunopathological changes during COVID-19 is pivotal for the identification of therapeutic targets. Our goals were to review the available literature concerning how the host immune status becomes unbalanced in COVID-19 and reveal the underlying mechanisms of the pathogenesis of COVID-19, thereby contributing to the development of therapeutics and the optimization of therapeutic strategies.

## COVID-19: a combat between SARS-CoV-2 infection and natural host immunity

SARS-CoV-2 is a type of positive-sense single RNA virus (27.9–31 kb) that uses enzymes of host cells for viral invasion, such as angiotensin-converting enzyme 2 (ACE2) and aminopeptidase N (APN) [11]. ACE2 receptors are not only highly expressed in alveolar epithelial cells, but also in other types of organs or tissues, such as those in the respiratory tract, gastrointestinal tract, adipose tissues, and vascular endothelial tissues [11]. Thus, the manifestation of SARS-CoV-2 infection is ranging from asymptomatic illnesses or mild flu-like symptoms to severe pneumonia and acute respiratory distress syndrome (ARDS).

**Figure 1.**
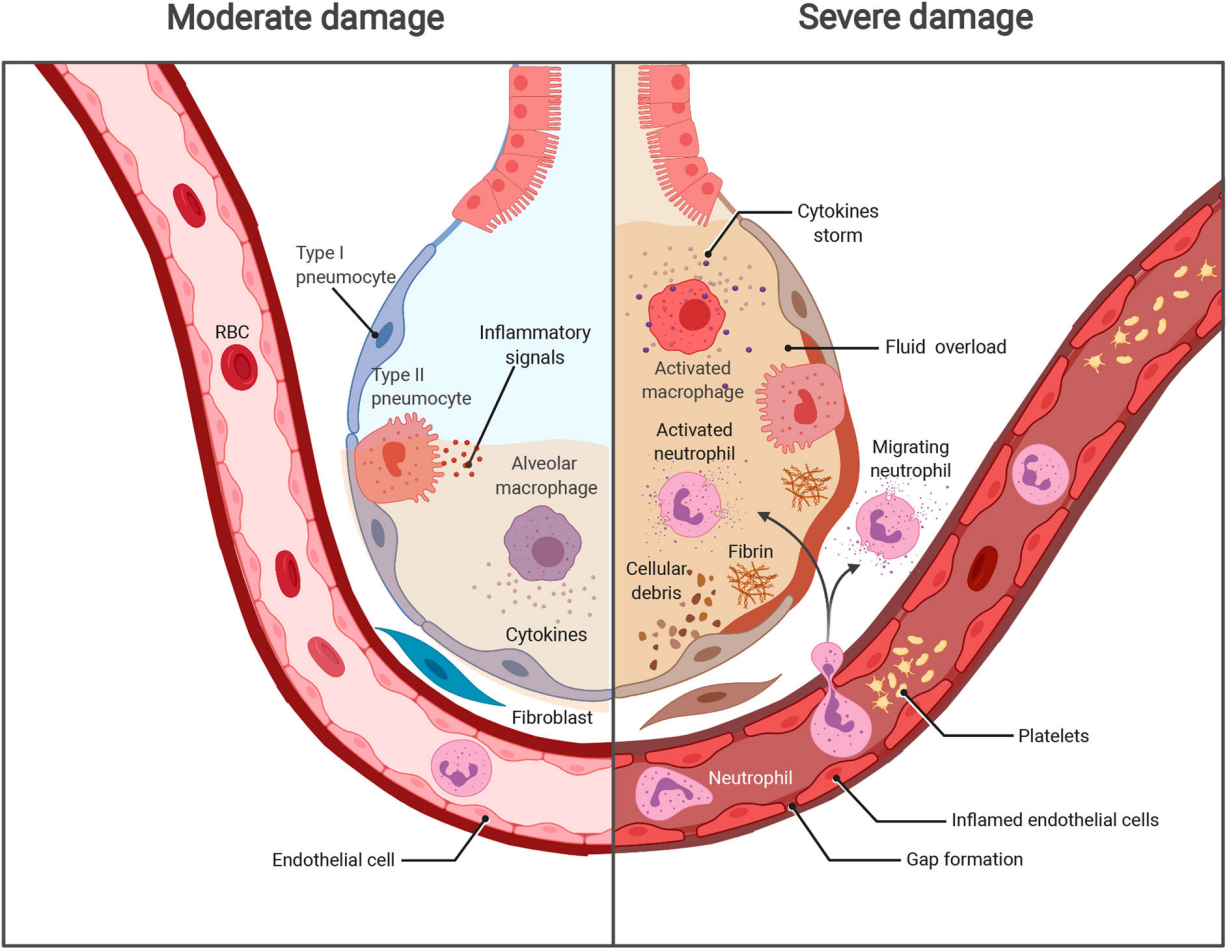
The progression of COVID-19 and potential damage. Left, SARS-CoV-2 infects the human airway epithelium via ACE2 receptors, which are mainly located on type II pneumocytes. In mild disease, the recruitment of myeloid cells occurs without causing an inflammatory storm, and the virus is cleared. Right, in severe COVID-19, SARS-CoV-2 escapes from the immune system, penetrates the epithelium, and infects endothelial cells that induce cell debris. Severe COVID-19 involves diffuse alveolar damage, endotheliitis, and fluid overload associated with neutrophils originated cytokine storm and NETs. The image was created with BioRender.com.

Indeed, the virus typically causes mild or moderate symptoms during the incubation period; meanwhile, the host immune system elicits protective responses against infection [12]. However, the natural host immunity and the outcomes of SARS-CoV-2 infection vary with health status; age; and the presence of commodities [13]. If the host immune response in fighting against SARS-CoV-2 infection, including innate immune signalling activation and adaptive immune components, fails to clear the virus, the patient then enters a severe stage of disease with an acute onset of bilateral infiltrates, severe hypoxaemia, and fluid overload [14]. Thus, we focused mainly on how the trinity of COVID-19 components, including viral infection, immunity, and the inflammatory immunopathogenesis, interact with one another (Figure 1).

## Innate immune evasion induced by SARS-CoV-2 infection

The IFN response, as a critical component of the innate immune response, is the first line of host antiviral defense [9]. In contrast with early reports of undetectable levels of type I and III IFNs in the serum of COVID-19 patients, emerging data obtained from those with severe COVID-19 offer evidence of a robust IFN-I responses after the initial IFNs delay. The delayed IFN type I response could enhance hyper-inflammation in the late stage of severe COVID-19 [15]. It is also demonstrated that SARS-CoV-2 widespread tropism for nasal epithelial cell types, resulting in the type I and III IFNs response in the nasal airway. The interferon response is notably delayed in onset relative to viral gene expression and obviously insufficient to control the virus [16].

As a critical immune-escape strategy, SARS-CoV-2 uses a variety of methods to antagonize the IFN system with the clinical consequence of an insufficient type I IFN response, which was documented in early COVID-19 studies [7,17]. SARS-CoV-2 infection disrupts the feedback of the IFN pathway by promoting the production of cellular ISGs that inhibit the IFN type I response, such as inhibitors of cytokine signalling proteins (SOCS3) [18]. SOCS3 strongly suppress TLR7-mediated IFN type I production by binding to IRF7. Moreover, SARS-CoV-2 has its own anti-IFN proteins [19]. One of the first known anti-IFN viral protein was called non-structural protein 1 (nsp1), which binds to the host ribosome and blocks the messenger RNA entry channel, thereby inhibiting cellular translation but not viral translation. Other viral proteins, such as nsp8, nsp9, and nsp16, are produced in the early stages of the viral life cycle before the production of double-stranded RNA. Alternatively, the induction of IFN could also be affected in other standby, neighbour, or older host cells, therefore causing inflammation and a wide degree of damage in the lungs [20].

## An imbalance in immune cell counterparts and its pathogenesis in COVID-19

### Neutrophilia, lymphopenia, and the value of the NLR related to the severity of COVID-19

Inflammatory cytokine storm is a typical laboratory abnormality observed in severe pathogenic coronavirus infections [21]. Great interest has arisen regarding on how the host immune system triggers cytokine storm in severe COVID-19. Numerous reports on blood vessel and pathological autopsies of COVID-19 patients have noted the tissue infiltration of neutrophils, which affect the pulmonary capillaries, acute capillaritis with fibrin deposition, and neutrophilic mucositis [10,22–24].

Lymphopenia is another critical feature of severe COVID-19 [4–6,25,26]. Herein, Liu et al. first reported the ratio of neutrophils to lymphocytes (NLR) in 61 COVID-19 patients and claimed that a higher NLR was a most potential predictor for the prognosis of severe COVID-19 [4]. Besides, the dynamic composition changes of lymphocytes and CD8^+^T-cells were clarified as the primary decreased lymphocyte subset in COVID-19 patients as mentioned before [6]. Hence, the ratio of neutrophils to CD8^+^ T-cells (N8R) was referred to as a factor comparable to NLR for receiver operating characteristic curve verification. Immunological markers involving N8R and NLR could serve as potential predictors for the progression and prognosis of COVID-19 [4].

Expect for unbalanced cell compartments, the state of immunosuppression was accompanied by a pathological state of hyper-inflammation, which was marked by elevated levels of cytokines, such as IL-6 and IL-8, also occurs in COVID-19, especially in moderate and severe disease [27]. Moreover, extensive infiltration of neutrophils and monocytes in the lungs is highly correlated with the severity of pathological damage during COVID-19 [5,28]. However, how the innate immune cells cause the state of hyper-inflammation and have an impact on the immunopathology of COVID-19 is not understood.

### NETs play a crucial role in viral clearance and immunopathology of COVID-19

The regulatory role of neutrophils in host immunity mainly includes phagocytosis, degranulation, and the release of neutrophil extracellular traps (NETs). Over the past several decades, the study of neutrophilic inflammation against pathogens concerned the production of NETs. NETs are large, extracellular, web-like structures composed of extracellular webs of DNA, histones, microbicidal proteins, and oxidative enzymes [29]. On the one hand, NETs can neutralize and kill pathogens; on the other hand, NETs mediate pathological inflammation in virus- or bacteria-infected tissues or organs. As mentioned, aggressive neutrophil activation, which directly leads to tissue damage with the procedure, such as oxidative bursts, phagocytosis, and the continuous generation of neutrophil NETs, is known as NETosis [29]. The NETosis process caused by a virus is a double-edged sword. Excessive NETosis damages the epithelium in pulmonary fungal infection and the endothelium in transfusion-related acute lung injury [30,31]. However, less is known about the role of neutrophils in COVID-19.

During the initial stage of the COVID-19 pandemic, serum markers of NETs – myeloperoxidase (MPO)-DNA, and citrullinated histone H3 (Cit-H3) – were first reported to be elevated in the serum of COVID-19 patients [5]. Veras et al. investigated the potential impact of NETs on the pathology of 32 hospitalized patients with severe COVID-19. The elevated levels of NETs in both tracheal aspirate and plasma, and a more pronounced concentration of NETs were found [32]. In addition, soluble NET markers were previously reported to be elevated in the BALF and alveolar spaces of patients with ventilator-associated pneumonia [28].

To better known the triggers and consequence of neutrophils’ activation and NETosis. Leppkes et al. identified platelet and complement activation as possible triggers of the formation of NETs in pulmonary and renal microcirculation [33]. Recent findings also suggest that SARS-CoV-2 can directly stimulate neutrophils to release NETs in a dose-dependent manner through ACE2 receptors [34]. In addition, Yaqinuddin et al. reported that cytokines such as IL-1β stimulation the formation of NETs in COVID-19 patients [10,35]. In addition, SARS-CoV-2 infection induces hypoxia and tissue inflammation, which in turn contribute to the release of NETosis and HMGB1 [36].

**Figure 2.**
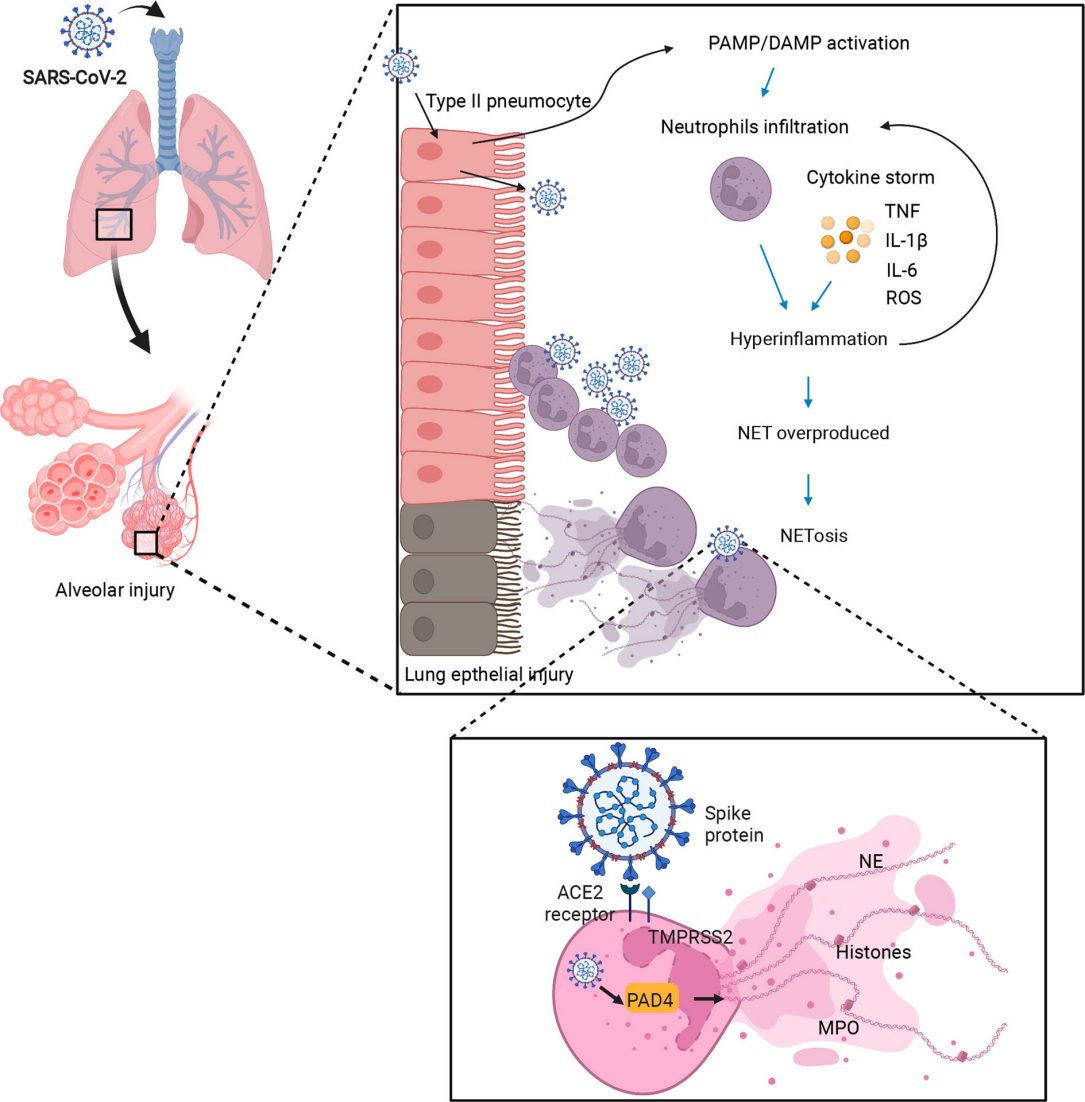
The hypothesis of how NETs lead to lung damage in COVID-19. First, SARS-CoV-2 infects the human airway epithelium via binding to ACE2 or TMPRSS2 receptors by invading type II pneumocytes. Insufficient PAMP/DAMP recognition is induced by viral evasion strategies and leads to inflammatory cytokine release. The inflammatory cytokines recruit neutrophils into viral-infected tissues in the lungs. The excessive inflammatory infiltration causes a cascade of cytokines (e.g. TNF-α, IL-6, IL-8), also called hyper-inflammation. Neutrophils involved in cytokine storm, on the other hand, lead to NET overproduction, represented by target proteins such as PAD4 as well as NE, priming the procedure of NETosis. Finally, the lung epithelium is injured by the processes of NETosis, reactive oxygen species, and myeloperoxidase overaction. The image was created with BioRender.com.

The ineffective attempts of NETs to clear a virus result in the catastrophe of cytokine storm, which is a cause of severe COVID-19 pneumonia [32–34]. To elaborate on the neutrophil’s role in immunology and the immunopathology of neutrophils in COVID-19, an in-depth investigation into the dynamic neutrophil response to explain the dynamic progression of COVID-19 was proposed. It’s mentioned that the sophisticated cascade of interactions in a severe COVID-19 case begins with the virus’ innate immune recognition but insufficient viral elimination, in which the IFN response as mentioned was involved. Following viral recognition, inflammatory cytokines are released [35]. Thereafter, neutrophils are activated to recruit inflammatory cells, and this can finally lead to the overproduction of NETs and an overload of viral replication, which is known as cytokine storm or hyper-inflammation. In addition to the damage of lung tissues induced by NETosis, the level of reactive oxygen species may also cause excessive activation of inflammatory pathways, such as the NF-κB pathway, exacerbating the tissue damage by hyper-inflammation and leading to life-threatening respiratory complications in severe cases of COVID-19 (Figure 2) [36].

### Role of monocytes/macrophages in COVID-19 pathogenesis

Circulating monocytes and monocyte-derived macrophages play another pivotal role in the host immunity to defend against SARS-CoV2 infection [28]. BALF from severe COVID-19 patients contains high levels of CC-chemokine receptor ligand (CCL2 and CCL7), which are most effective in the recruitment of 2-positive (CCR2^+^) monocytes [37]. Moreover, the population of classical inflammatory monocytes – CD14^+^CD16^+^ subsets that produce IL-6 – was significantly elevated in COVID-19 patients hospitalized in the intensive care unit compared to those patients who were not [38,39]. Of note, single-cell RNA sequencing (scRNA-seq) analysis of BALF collected from patients with severe or mild COVID-19 showed an increase in the proportion of mononuclear phagocytes, accounting for 80% of the total number of BALF cells. Besides, mononuclear phagocyte compartment alterations, were mainly manifested by a loss of tissue-resident alveolar macrophages and an infiltration of inflammatory monocyte-derived macrophages in severe cases [40].

**Figure 3.**
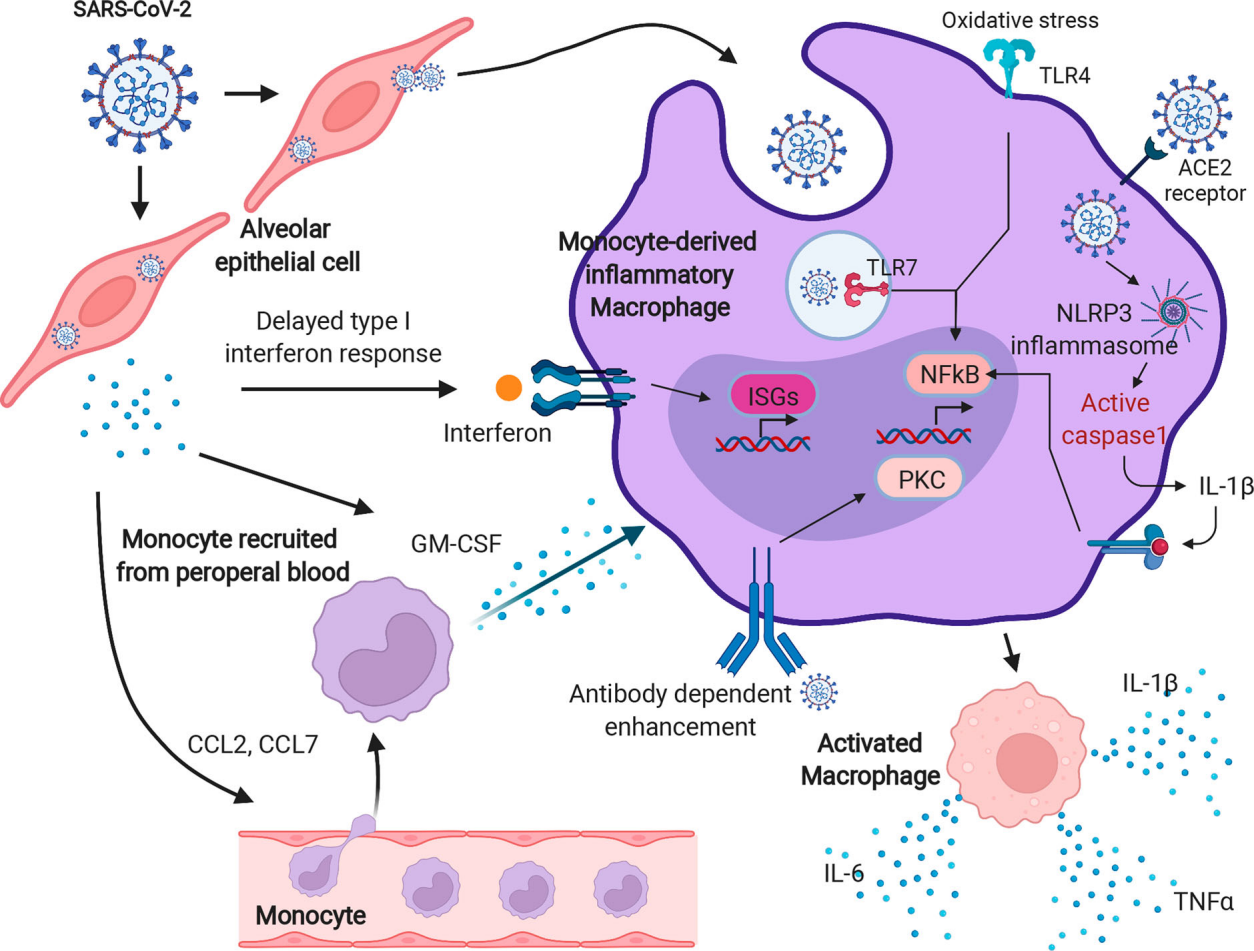
The hyperactivation of monocyte-derived macrophages in COVID-19. After SARS-CoV-2 infection, a delayed IFN response is induced, owing to the anti-IFN response triggered by the viral protein components. The insufficient IFN response leads to a failure of viral clearance by innate immunity, causing a recruitment of circulating blood cells, such as monocytes, and chemokine release. Moreover, those released chemokines, including CCL1 and CCL7, further provoke the monocytes into an inflammatory state, known as monocyte-derived inflammatory macrophages in the lungs with sustained PAMP/DAMP activation and NLRP3 inflammasome formation. These types of cells kill the lung epithelia either via the NLRP3 inflammasome activated-caspase pathway or oxidative stress reaction and enhance the cytokine storm by antibody-dependent enhancement. The image was created with BioRender.com.

Explanations regarding the excessive activation of monocyte-derived macrophages in patients with severe COVID-19 were exhibited. The delayed production of type I IFN, which leads to a failure to sense the virus, could enhance the release of chemokines and granulocyte-macrophage colony-stimulating factor (GM-CSF) from alveolar epithelial cells and result in a continuous recruitment of circulating monocytes into the lungs. Thereafter, monocytes differentiate into pro-inflammatory macrophages by activating the signal transducer and activator of the transcription (STAT) pathway [28]. Additionally, monocyte-derived macrophages could also be re-activated through Toll-like receptor pathways, such as TLR4 and TLR7, which finally leads to the overproduction of oxidative stress reactions in pathogen-infected lung tissues [41,42]. The immunopathological cascade evoked by monocytes and macrophages could also occur owing to the upregulation of SARS-CoV-2 entry receptors. It is plausible that type I IFN induces the expression of SARS-CoV-2 entry receptors, such as ACE2 and CD147, facilitating the virus to gain access to the cytoplasm of macrophages and to activate the NLRP3 inflammasome, leading to increased secretion of mature IL-1β [28]. The mechanisms mentioned above, therefore, point directly to the formation of COVID-19 cytokine storm by the accumulation of large amounts of pro-inflammatory cytokines (Figure 3).

### Alterations of lymphocytes and NK-cells in COVID-19 cases

Lymphopenia is ubiquitous in COVID-19 patients with significantly decreased absolute counts of T-cells, and increased levels of pro-inflammatory cytokines (e.g. IFN-γ, IL-6, IL-8) [43,44]. In addition, T-cells are critical for suppressing the overactive innate immune response during viral infection [45,46]. Therefore, the loss of T-cells during SARS-CoV-2 infection may lead to increased inflammation. Consistent with the above conception, a T helper2 (Th2) immune response was detected in peripheral blood, manifesting as a high proportion of basophils, degranulated eosinophil’s and Th2 cytokines (e.g. IL-4 and IL-10) [47]. Additionally, more pro-inflammatory CCR6^+^ Th17 subsets in the peripheral blood of severe COVID-19 cases than mild COVID-19 cases were reported [48]. Sadeghi et al. mentioned that a raised Th17 cell response and a reduced function of Treg cells, respectively, caused a hyperinflammation and the disease progression of COVID-19, particular in the dead COVID-19 cases. The elevated ratio of Th17/Treg, the accentuated Th17-relevanted factors (ROR-γt, IL-17, and IL-23), and the decreased Treg-associated factors (FoxP3, TGF-β, and IL-10), may play a critical role in increasing the inflammatory responses and the disease pathogenesis in COVID-19 patients [49]. Controversially, other studies noted that increased Treg proportions and activation status correlated with COVID-19 severity [50,51]. A striking phenotype perturbation of Tregs showing the over-expression of pro-inflammatory cytokines, such as Treg COVID-19 phenotype-inducers, interleukin (IL)-6 and IL-18 were found in severe COVID-19 cases compared with mild cases, recovered patients, and healthy donors [51]. Thus, we speculated a dynamic perturbation of Treg cells in COVID-19 progression. The decrease ratio of Treg might be closely related to the pathogenesis of COVID-19, particularly during the phase of cytokines storm. Whereas the ratio of Treg might be elevated in severe COVID-19 cases owing to the exhaustion of immune system, particularly those ICU hospitalizations.

Not only dramatically reduced CD4^+^ and CD8^+^T-cell counts in severe COVID-19 cases but also the dampened functionality of T cells was detected. For example, activation markers such as HLA-DR, CD45RO, and CD38 and the exhaustion markers PD-1, Tim-3, and NKG-2A on T-cells are highly expressed, which indicates that T-cells are dysregulated without specific antiviral function [52]. In our previous study, we also proved that T-cells are negatively regulated by neutrophils in severe COVID-19 cases [5]. Approximately 80% of mild-to-moderate COVID-19 cases can fully recover at home. However, most convalescent plasma samples of recovered COVID-19 patients did not show high concentrations of neutralizing activity or enough antibodies specific to viral proteins to suggest effective antiviral activity [53]. Consistent with this finding, Johannes et al. further investigated the SARS-CoV-2 spike–specific B-cell responses in 14 recovered COVID-19 patients using scRNA-seq. Their in-depth analysis showed distinct B-cell subsets transcriptionally but a low binding and non-neutralizing monoclonal antibody in convalescent individuals [54].

**Figure 4.**
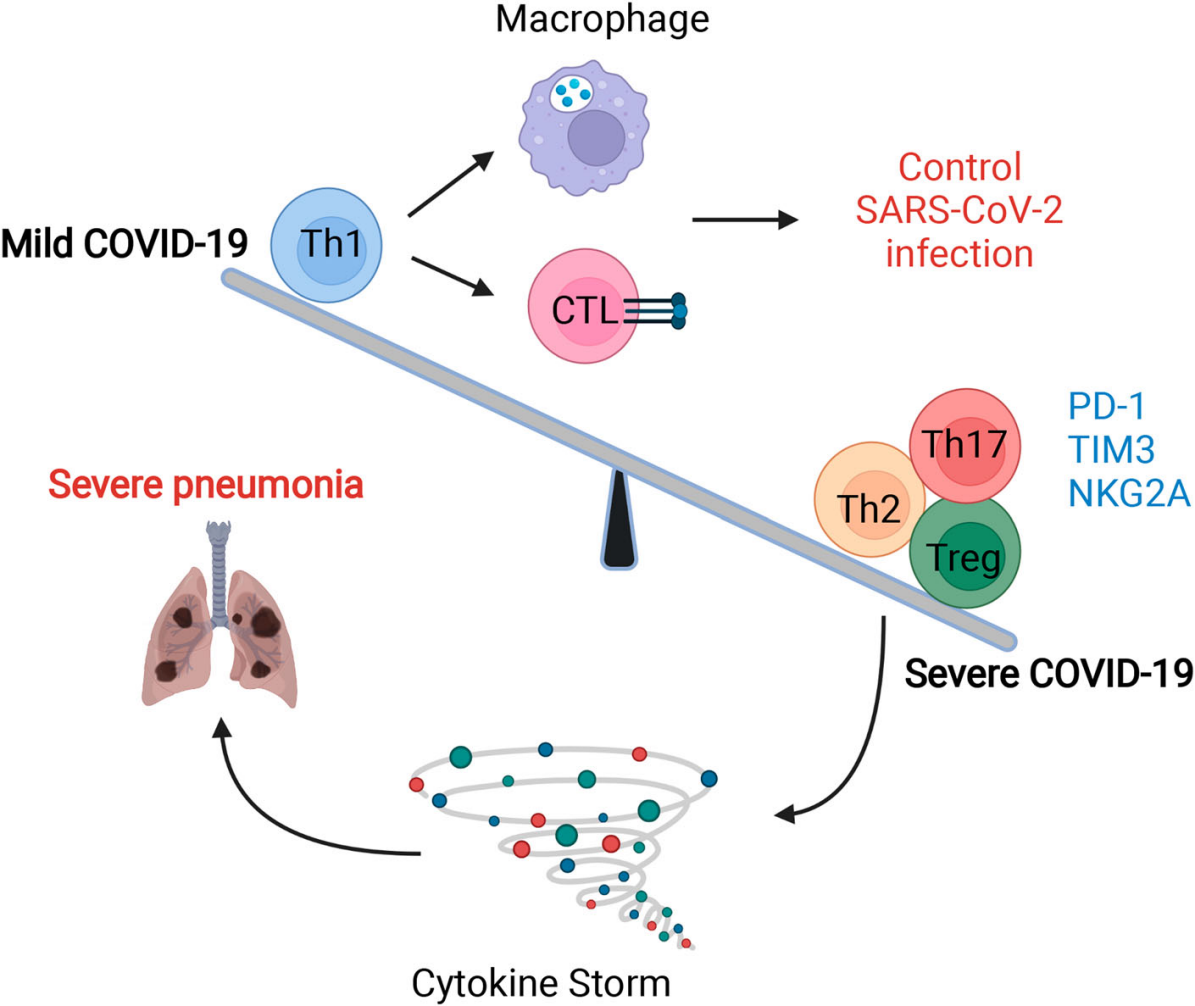
The imbalance in T-cell immunity between severe and mild COVID-19. The dysregulation of T-cells includes imbalanced immune cell compartments and the expression of inhibitory molecules such as PD-1, Tim-3, and NKG-2A. The dynamic disease progression from mild to severe symptoms, which manifested with cytokine storm, was exhibited with distinct T subsets and T-cell responses. In mild COVID-19 cases, the Th1 subsets were higher than those in severe COVID-19 cases, leading to a strong CTL and macrophage phagocytes. However, in severe cases, Th2, Th17, and Treg subsets were comprised mainly of T-cells, together also with highly expressed inhibitory molecules, such as PD-1, TIM-3, and NKG2A. The image was created with BioRender.com.

Meanwhile, in the natural process of SARS-CoV-2 infection, studies have also argued that the size of the anti–SARS-CoV-2 immunoglobulin G titre is closely related to the breadth of circulating virus–specific T-cell responses [55–57]. Except for the stem-like memory phenotype, the strong T-cell response to SARS-CoV-2 targets the spike (S) surface glycoprotein, owing to an increase in effector and Th1 subsets and Th1 cytokines in mild and moderate patients but reduced inhibitory T-subsets, such as Th2 and Treg cells, in severe COVID-19 patients (Figure 4). Notably, our previous study further detected sustained SARS-CoV-2–specific T-cell immune responses rather than neutralizing antibodies from COVID-19 patients within 7 months, which further implies a strong protective immune effect of T-cells [58].

As is known, the main function of the B-cell response against SARS-CoV-2 and SARS-CoV-2 variant infection is to cross-neutralize the virus, while the humoral immune responses to COVID-19 vaccination constitute another complex situation not systematically described here. Briefly, viral antibody-dependent enhancement (ADE), which could be enhanced by defective or ineffective response to vaccination to facilitate the activation of inflammatory cells, such as monocyte-derived macrophages, thereafter, leads to the hyperactivation of monocyte-derived macrophages in the lung tissues of COVID-19 patients [59–62]. However, an effective COVID-19 vaccination could also accelerate cellular immune responses, manifested in T and B cell response; provide protective immunity; and reduce pulmonary complications [63]. T cells and B cells were supposed to be reactivated and retained as immunological memory cells against reinfection with SARS-CoV-2. For example, Moderna mRNA-1273, Pfizer/BioNTech BNT162b2, Janssen Ad26.COV2.S, and Novavax NVX-CoV2373 induced memory CD4^+^T cells for 100% of individuals which lasts at least 6 months. Importantly, mRNA or NVX-CoV2373 vaccination also led to highly host Tfh and CD4-CTL response. mRNA and Ad26.COV2.S immunization induced optimistic CD8^+^T cell levels, which was detectable in 60%–67% of subjects at 6 months. Ad26.COV2.S immunization accompanied with a high frequency of CXCR3^+^ memory B cells [64]. Overall, these findings suggest that a strong and extensive T-cell response, rather than B-cell response could be efficient to provide immune protection against SARS-CoV-2 naturally.

Other than lymphocytes, we also noticed the alterations of other cell types, particularly NK cells in COVID-19 cases. Similarity to the perturbation of lymphocytes, NK cells were also dramatically reduced in severe COVID-19 cases compared to mild cases and healthy individuals. The reduced peripheral blood NK cell levels, particularly the cytotoxic CD56^dim^cells, and their functional exhaustion may directly lead to the progression and severity of COVID-19 [65]. Indeed, the phenotype of exhausted NK cells from COVID-19 cases was represented with a higher expression of inhibitory markers, such as PD1, Tim-3, NKG2A, LAG3, PDCD1, and HAVCR2 on NK cells, leading to a decreased expression of IFN-γ, IL-2 and TNF-α as well as reduced granzyme B levels via inflammatory regulating pathway, like NF-κB-dependent upregulation of ICAM-1 expression in target cells [66–68]. Additionally, NK cells, particularly the inflammatory NK subsets are involved in the cytokines-induced lung pathology. They were recruited to inflamed site of lungs by chemokines MCP-1 and IP-10, while the effector function of NK cells was impaired [69].

## An update on innate immune and T-cell evasion strategy for SARS-CoV-2 variants

Although the emergence of SARS-CoV-2 variants implies better viral adaptations to host immune pressures, less is known about SARS-CoV-2 variants’ impact on innate immune evasion and T-cell response [70]. Herein, we focus on explaining the problem. For example, B.1.1.7 antagonizes host innate immune responses more effectively than the original virus through upregulating subgenomic RNA and protein synthesis of key innate immune antagonists, such as open reading frames 9b and 6b (Orf9b and Orf6) [71].

Consolidated evidence suggests that T-cell–mediated immune responses are critical for immunity from SARS-CoV-2 infection. SARS-CoV-2–specific T-cells are essential for viral clearance, even without seroconversion. T-cell–mediated immunity provides robust memory after the natural infection process or vaccination [72]. However, Jordan et al. identified almost equivalent T-cell responses to VOCs (Alpha and Delta) and vaccination with BNT162b2 [73]. Most recently, Caniels et al. also declared that SARS-CoV-2 variants did not evolve further to escape from T-cell–mediated immunity [74]. As reported, the recognition of viral variants also relies on cellular memory immunity, especially MHC-I involved cytotoxic T lymphocyte (CTL) response. These authors recorded the downregulated human leukocyte antigen (HLA)-A, -B, and -C genes’ messenger RNA expression in both the ancestral USA-WA1 strain, as well as the Alpha and Beta VOCs. Since SARS-CoV-2 open reading frame 8 (ORF8) induces autophagic degradation of the major histocompatibility complex class I (MHC-I) molecule, SARS-CoV2 variants escaping from cytotoxic T lymphocyte (CTL) surveillance are modulated by the ORF8 protein. Notably, despite unique mutations of ORF8 genes in SARS-CoV-2 VOCs, they are unable to suppress MHC-I expression currently [75]. Herein, it is plausible to speculate that the combination of prior infection and effective vaccination could contribute to shutting down MHC-I degradation, therefore evading the future viral evolution.

## Immunological and pathological mechanisms, targets for potential therapeutic strategies

Overall, the host immune system reacts to SARS-CoV-2 dynamically, while overreaction leads to the pathological damage of virus-infected tissues and organs. In the initial stage of SARS-CoV-2 infection, the virus enters bronchial epithelial cells and induces a type I IFN response [9,15]. Then, inflammatory signalling molecules are released from infected cells, followed by the recruitment of circulating blood cells. Thereafter, the SARS-CoV-2 virus further infects pulmonary capillary endothelial cells and triggers an influx of monocytes and neutrophils [32,33,39]. Meanwhile, excessive neutrophils with NETosis activate macrophages from blood monocytes instead of resident alveolar macrophages and induce increasing inhibitory T-cell subsets and dysfunction of viral-specific T-cell responses [29,39,48–52]. Neutrophils and NETs further have an impact on the hyper-inflammation, coagulopathy, endothelial dysfunction, and immunothrombosis in severe COVID-19 cases [32–36]. The imbalanced immune response together contribute to cytokine storm, pneumonia, and viral sepsis.

**Figure 5.**
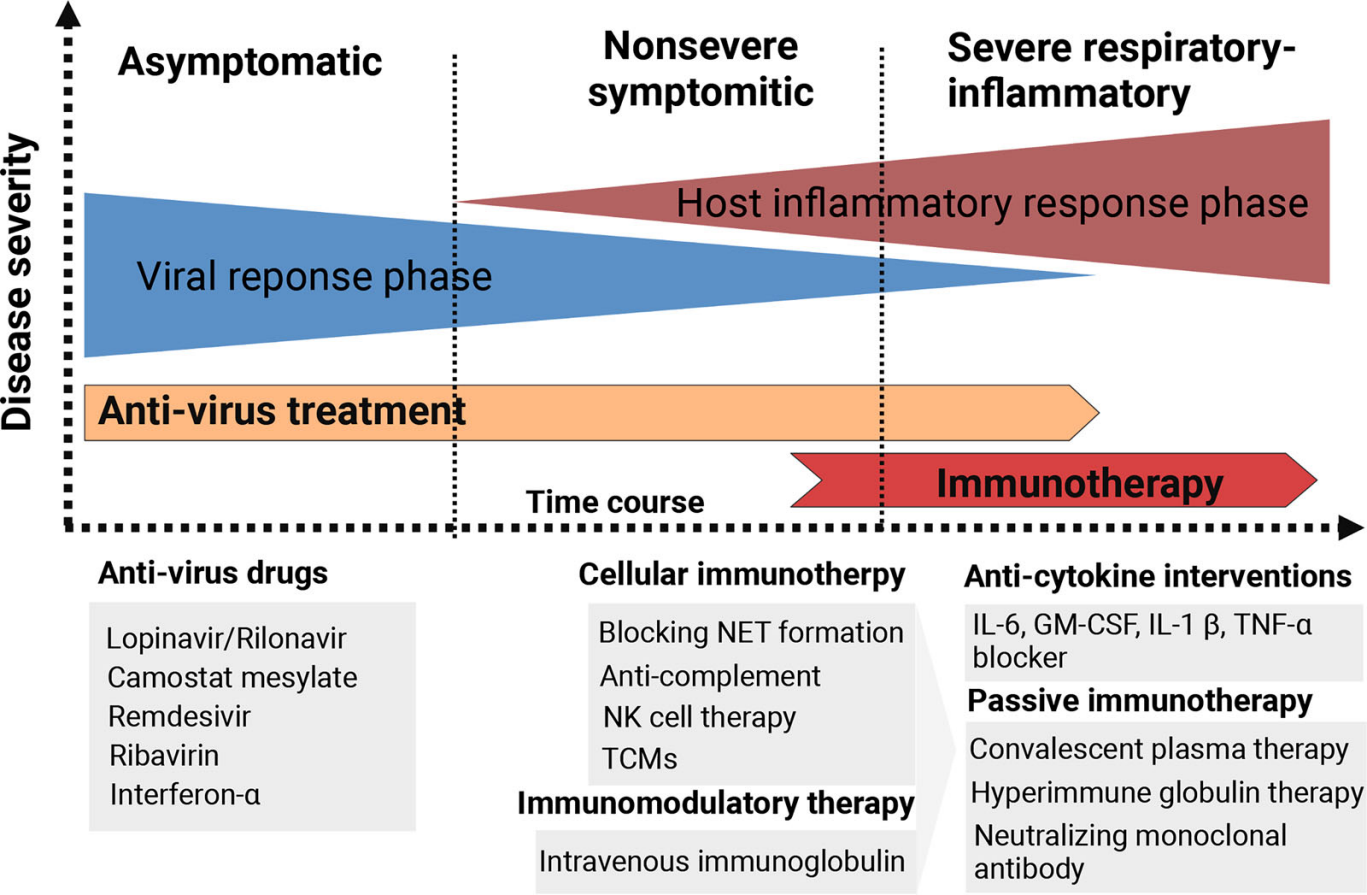
Different immunotherapy strategies for COVID-19 patients. Anti-virus drugs and multiple types of immunotherapeutic targets against severe COVID-19 patients are illustrated. The image was created with BioRender.com.

Immunological and pathological mechanisms provide various targets for potential immune therapeutic strategies (Figure 5). For example, blocking NET formation by *glucocorticoids* is likely to be an effective therapeutic strategy [76]. Moreover, since ADE induced macrophage activation, anti-complement strategies based on an anti-C5/C3 humanized monoclonal antibody (eculizumab or AMY-101) have successfully been used in a small proportion of severe patients [77]. Besides, the use of IL-6 blocker (i.e. tocilizumab), and GM-CSF blocking were verified to be useful in a small proportion of severe COVID-19 patients by attenuating their hyper-inflammation and cytokine storm [78]. Recently, NK cell therapy CYNK-001 in COVID-19 patients was permitted by FDA. CYNK-001 included CD34+ stem-cell-derived NK cells and were culture expanded. In addition to NK cell therapy, other cell-based therapies, like SARS-CoV-2-specific T cell therapies, are also considered [79]. Additional immunotherapies for COVID-19 of which have broad anti-inflammatory effects, such as pooled normal immunoglobulin G (IgG) or intravenous immunoglobulin (IVIG) therapy. Passive immunization of SARS-CoV-2 by using convalescent plasma, intravenous hyperimmune globulin (high concentrations of neutralizing antibodies obtained from recovered patients), or neutralizing monoclonal antibodies could be also potential therapeutic options [80].

But whether immune dysregulation drives COVID-19 pathology is controversial, since clinical reports on patients taking immune-modulating medications show no advantages for individuals not receiving such medications [81]. Likewise, the effect of cytokine blockage, such as that achieved with tocilizumab, was reported to be only effective in a small portion of COVID-19 patients. Our review revealing the immunological and pathological mechanisms in COVID-19 is beneficial to explain these problems and supply clues for optimizing immune therapeutic strategies. We emphasized the dynamic procedure of disease progression, which suggests the importance of appropriate timing for immunotherapeutic strategies. For example, we support early IFN treatment for COVID-19 to promote innate evasion [82]. An optimal benefit can be obtained with anti-IL-6/JAK therapies, and the use of tocilizumab/ruxolitinib were suggested during a finite window of opportunity at the outset of hyper-inflammation but before fulminant disease causes irreversible tissue damage [83]. Corticosteroids is one of another crucial type of immunotherapeutic approaches for SARS, while controversial for the treatment of COVID-19. Hajar et al, reviewed different COVID-19 prognosis after using Corticosteroids during distinct clinical stages of disease. They eventually pointed that COVID-19 patients on mechanical ventilation or COVID-19 patients with respiratory failure (ARDS) were benefited from dexamethasone treatment. Nevertheless, they mentioned others’ considerations regarding to the use of corticosteroids that the statue of patients and the pros and cons of corticosteroids treatment should be carefully evaluated before corticosteroids administration [84].

SARS-CoV2 and its variants induce different levels of immunopathology, which leads to different symptoms. Therefore, individual therapeutic strategies should be chosen according to the distinct symptoms of COVID-19 patients. Finally, it’s proposed that the critical COVID-19 is a doubly deficient disease requiring both a defective of viral control through impaired type I and type III IFN systems and the subsequently unbalanced adaptive immune regulation and pro-inflammatory activity. The critical COVID-19 develop only when all checkpoints fail. Vice versa, owing to the existing of self-limiting of host immunity, once the limitation step is breached by SARS-CoV2 infection, there will be a cascade effect that is difficult to correct [85]. Thus, a combination of immunotherapeutic strategies may better be able to overcome COVID-19-related cytokine storm and hyper-inflammation than monotherapy if required [86]. Due to the urgent need to develop a broad-spectrum inhibitory small molecule with potent antiviral capacity against SARS-CoV2 and its variants, it is useful to optimize the combination of antiviral agents and immunotherapeutic approaches with more clinical trials.
